# Astragaloside Inhibits Hepatic Fibrosis by Modulation of TGF-*β*1/Smad Signaling Pathway

**DOI:** 10.1155/2018/3231647

**Published:** 2018-04-30

**Authors:** Xingxing Yuan, Zhiqiang Gong, Bingyu Wang, Xueying Guo, Lei Yang, Dandan Li, Yali Zhang

**Affiliations:** ^1^Department of Gastroenterology, Nangang Branch, Heilongjiang Academy of Traditional Chinese Medicine, No. 33 West Dazhi Road, Nangang District, Harbin, Heilongjiang 150006, China; ^2^School of Pharmacy, Faculty of Chinese Medicine Science, Guangxi University of Chinese Medicine, No. 13 Wuhe Road, Qingxiu District, Nanning, Guangxi 530222, China; ^3^Pharmacology Laboratory of Traditional Chinese Medicine, Heilongjiang Academy of Traditional Chinese Medicine, No. 41 Xiangshun Road, Xiangfang District, Harbin, Heilongjiang 150036, China

## Abstract

Activation of HSC is a pivotal step in hepatic fibrosis. In the activation of HSC, the TGF-*β*1 plays a key role that can promote the occurrence of hepatic fibrosis by combining with Smad proteins. Astragaloside is the main active component extracted from* Radix Astragali* that has the effect of antioxidation and hepatoprotection. In the present study, we investigated the mechanism of astragalosides inhibiting hepatic fibrosis* in vitro* and* in vivo*.* In vitro*, astragalosides inhibited the activation of HSC and regulated the expression of MMP-2 and TIMP-2 and reduced the formation of collagen fibers.* In vivo*, administration of astragalosides decreased the serum ALT, AST, and TBiL in rats by reducing oxidative stress. Astragalosides also attenuated hepatic fibrosis by reducing the concentration of hydroxyproline and inhibiting the formation of collagen fibers. The expressions of TGF-*β*1, T*β*R-I, p-Smad 2, and p-Smad 3 were downregulated after astragalosides treatments, while Smad 7 was upregulated compared to the control group. The results indicated that the effect of astragaloside on hepatic fibrosis was related to the inhibition of HSC activation and the modulation of the TGF-*β*1/Smad signaling pathway.

## 1. Introduction

Hepatic fibrosis is the common stage in the development of a variety of chronic hepatic diseases, characterized by atypical hyperplasia of intrahepatic connective tissues and excessive accumulation of extracellular matrix (ECM) [1, 2]. Therefore, it is important to protect hepatic cells and reduce the formation of collagen fibers during chronic hepatic diseases. A wide array of factors such as chronic hepatitis viral infection, fatty liver, alcohol, and drug-induced liver injury can induce the production and activation of hepatic fibrosis-initiating cells, such as hepatic stellate cells (HSC), fibroblasts, liver sinusoidal endothelial cells, and hepatocytes [3, 4]. These cells can proliferate and transdifferentiate into myofibroblasts and then synthesize and secrete a large amount of ECM, which leads to hepatic fibrosis and cirrhosis [5]. Clinical treatment of hepatic fibrosis includes the elimination of pathogenic factors and reduction of ECM accumulation. However, the efficacy of current therapies is still limited.

The dormant HSC cells, once activated, transform into *α*-smooth muscle actin- (*α*-SMA-) positive myofibroblast-like cells that regulate the expression of fibrillar collagen and metalloproteinase inhibitors (TIMPs) such as TIMP-1 and TIMP-2, as well as metalloproteinases (MMPs) such as MMP-2 and MMP-9, which are the major contributors to ECM development [6–8]. Due to the important role of HSC in the formation of ECM, the activation of HSC is considered to be an essential part of hepatic fibrosis. Transforming growth factor *β*1 (TGF-*β*1) has been confirmed to be the main cytokine in the generation of reactive oxygen species (ROS) and ECM production, which can stimulate the activation and proliferation of HSC by activating its downstream Smad signaling pathway [9, 10]. Therefore, the TGF-*β*1/Smad signaling pathway is considered a potential target for the prevention and treatment of hepatic fibrosis.

An increasing amount of natural compounds has been proved to be hepatoprotective.* Radix Astragali* (the root of* Astragalus*; Huangqi),* Astragalus membranaceus*, and its variety Mongolia are commonly used species. They have been used for more than 2,000 years as a herbal medicine in China for hepatoprotection and antifibrosis [11]. Astragaloside (AST), the major active components of* Radix Astragali*, consists of several triterpene saponins, such as astragalosides I–IV [12]. A large amount of evidence has proved that AST has a wide range of pharmacological effects, including antioxidant stress, anti-inflammatory, anticancer, and antifibrosis [13–16]. Furthermore, little side effect has been reported. However, the mechanism of AST on hepatic fibrosis is still not clear and required further investigation.

In this study, we investigated the antifibrosis mechanism in vitro and in vivo. In vitro, AST was added to cultured HSC induced by TGF-*β*1 to observe the interventions on proliferation and apoptosis. The expressions of COL-I, COL-III, MMP-2, and TIMP-2 were measured as indicators of the effects of AST on ECM. The hepatic fibrosis in vivo model was established by administrating carbon tetrachloride (CCl4) to rats that causes hepatic cell injury. Their lipid peroxidation levels were also examined. The results were compared with that of malotilate, a drug known to reduce the secretion of inflammatory factors and inhibit the activation of HSC, thereby inhibiting the synthesis and deposition of hepatic collagen and reducing the inflammatory response and fibrosis in the liver [17]. The expression of related components and their phosphorylation levels in the TGF-*β*1/Smad signaling pathway were also analyzed.

## 2. Materials and Methods

### 2.1. Reagents

HSC-T6 was purchased from Beijing Friendship Hospital (Beijing, China). TGF-*β*1, LY-364947 [4-[3-(2-pyridinyl)-1H-pyrazol-4-yl]-quinoline HTS 466284 transforming growth factor *β* Type I receptor kinase inhibitor, molecular formula: C17H12N4, weight: 272.30, and purity: ≥95% (HPLC)], DMSO, and MTT were purchased from Sigma (St. Louis, USA). AST [molecular weight: 248.36386; purity: ≥98%] was purchased from Pure-One Bio Technology (Shanghai, China). Malotilate was purchased from Yabang Epson Pharmaceutical (Jiangsu, China). Kits of ALT, AST, TBil, Alb, MDA, GSH, SOD, COL-I, COL-III, *α*-SMA, MMP-2, TIMP-2, hydroxyproline, H&E staining, Masson's trichrome staining, Sirius red staining, and Annexin V-FITC/PI double staining were purchased from Jiancheng Bioengineering Institute (Nanjing, China). Antibodies against *α*-SMA, TGF-*β*1, T*β*R-I, Smad 2, p-Smad 2 (S465/467), Smad 3, p-Smad 3 (S423/425), Smad 7, and *β*-actin were purchased from Cell Signaling Technology (MA, USA). HRP-labeled secondary antibodies were purchased from ZSGB-BIO (Beijing, China). RevertAid™ First Strand cDNA Synthesis Kit and SYBR Green Real-Time PCR Kit were purchased from Thermo Fisher (MA, USA).

### 2.2. Cell Culture and Treatment

The rat HSC-T6 cell line was cultured in DMEM medium containing 10% fetal bovine serum (FBS) with 5% CO_2_ at 37°C. The 3rd or 4th generations of the cells, when grew to approximately 80%, were digested with trypsin to enhance permeability. The powder form of each compound (2 mg) was added to 200 *μ*L DMSO until being completely dissolved; then 1800 *μ*L ddH_2_O was added to obtain a working solution of 1 mg/mL for use—grouping: control group; model group: TGF-*β*1 (1 ng/ml) group; AST (low dose group): TGF-*β*1 (1 ng/ml) + AST (12.5 *μ*g/ml); AST (medium dose group): TGF-*β*1 (1 ng/ml) + AST (25 *μ*g/ml); AST (high dose group): TGF-*β*1 (1 ng/ml) + MT (50 *μ*g/ml); TGF-*β* receptor 1 (T*β*R-1) inhibitor group: TGF-*β*1 (1 ng/ml) + LY-364947(2 *μ*M).

### 2.3. Detection of Cell Viability

The methyl thiazolyl tetrazolium (MTT) assay was performed in 96-well plates, and the inoculum was 5 × 10^4^ cells/ml. Experiments were performed with 5 replicates. Following 24 h of incubation after various treatments, 10 *μ*l of MTT solution at 5 mg/mL was added to 100 *μ*l culture media and cells were incubated for a further 4 h at 37°C. After removing the culture medium, 200 *μ*L DMSO was added to each well and then the plates were shaken for 10 min and the absorbance (OD) values were measured at A490 nm.

### 2.4. Detection of Cell Apoptosis

24 h after the various treatments, the cells were collected into EP tube and centrifuged at 4°C and 3000 rpm for 10 min. After being rinsed with phosphate buffered saline (PBS) twice, the cells were resuspended in 500 *μ*l of binding solution. After filtration, the cells were transferred into another tube for antibody labeling: 8 *μ*l of PI and 4 *μ*l of Annexin V-FITC were added at room temperature in darkness and the specimens were detected using a flow cytometer (BD Bioscience, CA, USA) in 5 min.

### 2.5. Animal Experiment Design

Seventy male SD rats (160–180 g) were obtained from Heilongjiang Academy of Traditional Chinese Medicine and the research was conducted according to protocols approved by Institutional Ethical Committee of Heilongjiang Academy of Traditional Chinese Medicine. Within the one-week acclimation period, rats were randomly divided into seven groups (10 rats/group) including the control group, CCl4 group, CCl4 + AST groups (low, medium, and high dose group), CCl4 + malotilate group, and CCl4 + LY-364947 group. Except the control group, rats were subcutaneously injected with 50% CCl_4_ mixed with peanut oil, twice a week for 8 weeks. AST (low dose, 20 mg/kg; medium dose, 40 mg/kg; and high dose, 80 mg/kg ig.), malotilate (90 mg/kg ig.), and LY-364947 (1 mg/kg ip.) were coinjected with CCl_4_. The control group was given the same volume of vehicle (distilled water and peanut oil). At the end of the treatment, all animals were sacrificed; blood and liver tissues were obtained for further examination.

### 2.6. Histological Examination

Liver tissues were fixed in 10% formalin, embedded in paraffin, and sectioned at 5 *μ*m in thickness. The liver pathology was observed by hematoxylin-eosin (H&E) staining. Additionally, the collagen deposition was revealed by Masson's trichrome staining and Sirius red staining.

### 2.7. Enzymatic Assays and Biochemical Reactions

Serum alanine aminotransferase (ALT), aspartate aminotransferase (AST), total bilirubin (TBil), and albumin (Alb) levels were measured by corresponding biochemical assay kits. 100 mg of wet liver samples was subjected to acid hydrolysis to measure the hydroxyproline content according to the hydroxyproline testing kit.

To measure the levels of malondialdehyde (MDA), glutathione (GSH), superoxide dismutase (SOD), collagen type I (COL-I), collagen type III (COL-III), *α*-smooth muscle actin (*α*-SMA), matrix metalloproteinase-2 (MMP-2), and tissue inhibitor of metalloproteinase-2 (TIMP-2), liver tissues were firstly homogenized with Tris-HCl. The homogenate was centrifuged at 4°C and 10,000 rpm for 10 min. Supernatants of HSC-6 cells were also collected. Measurements were done using corresponding biochemical assay kits.

### 2.8. Western Blot Analysis

Proteins from liver tissues and from HSC-T6 cells were harvested and protein was extracted in ice-cold lysis buffer (RIPA), containing protease inhibitor cocktail and phosphatase inhibitor (Sigma Chemical Co., USA). Protein concentration was determined using the bicinchoninic acid (BCA) method. 80 *μ*g of proteins was subjected to SDS-PAGE and transferred to PVDF membranes (Bio-Rad, America). The membranes were blocked with 3% BSA in PBS for 2 h and then incubated with primary antibodies with dilution factors in the brackets: anti-*α*-SMA (1 : 1000), anti-TGF-*β*1 (1 : 2000), anti-T*β*R-I (1 : 1000), anti-Smad 2 (1 : 1000), anti-p-Smad 2 (1 : 300), anti-Smad 3 (1 : 1000), anti-p-Smad 3 (1 : 1000), anti-Smad 7 (1 : 1000) antibodies, and HRP-labeled secondary antibodies. After being washed three times in PBS, the membranes were detected using an ECL kit (Thermo Fisher, USA). The expression levels of protein were quantified by* Image J* software with normalization to *β*-actin.

### 2.9. Real-Time PCR

Expressions of COL-I, COL-III, *α*-SMA, MMP-2, TIMP-2, TGF-*β*1, T*β*R-I, Smad 2, Smad 3, and Smad 7 mRNA were quantified by real-time PCR. The primer sequences were presented in Table 1. The Trizol method was utilized to extract total RNA from the HSC-T6 cell and liver tissues. cDNA was synthesized using 2.5 *μ*g of total RNA as template, and the reaction volume was 20 *μ*l according to the RT kit. Quantitative real-time PCR was performed on Applied Biosystems Inc 1900 system with the SYBR Green Real-Time PCR Kit. PCR was performed at 94°C for 1 min and 60°C for 1 min for 30 cycles after denaturation at 95°C for 5 min. Melting was obtained immediately after the reaction to analyze the specificity of the PCR reactions. Quantitative analysis of target gene expression data was based on 2^−ΔΔCt^ method.

### 2.10. Immunohistochemistry

Sections were deparaffinized in xylene and dehydrated through a graduated alcohol series. After incubating with 0.3% H2O2 in methyl alcohol for 15 min, sections were treated with citrate buffer (pH 6.0) for antigen retrieval and incubated with 10% normal goat serum in PBS for 1 hour at room temperature to block nonspecific binding. Then, the sections were incubated with primary antibodies in PBS at 4°C overnight. After equilibrating to room temperature, sections were first incubated with appropriate secondary antibodies for 2 hours and then treated with a streptavidin-horseradish peroxidase complex for 1 h at room temperature. Staining was done by incubation with 3,39-diaminobenzidine (DAB) and the sections were counterstained with hematoxylin and viewed under a light microscope.

### 2.11. Statistical Analysis

SPSS17.0 software was used to analyze the data of each group, and the result was expressed as mean ± standard deviation; significance of difference between groups was analyzed with ANOVA, and the LSD test was used in pairwise comparison among multiple groups, with *P* < 0.05 considered as significant.

## 3. Results

### 3.1. AST Inhibited Cell Viability and Promoted Apoptosis

**Figure 1:**
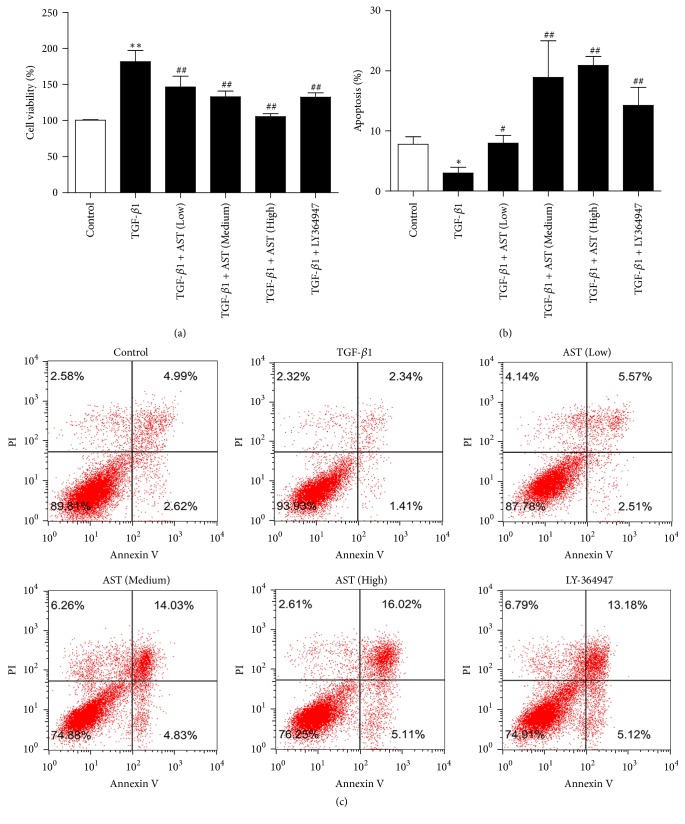
Effects of astragalosides on cell viability and apoptosis of hepatic stellate cells induced by TGF-*β*1. (a) By use of MTT, AST inhibited cell viability in a concentration-dependent manner. (b-c) By assay of flow cytometry, AST promoted apoptosis in a concentration-dependent manner. AST: astragalosides; LY-364947: TGF-*β* receptor 1 inhibitor. The values presented are the means ± SD (*n* = 5 in each group). ^*∗*^*P* < 0.05 and ^*∗∗*^*P* < 0.01 versus control group; ^#^*P* < 0.05 and ^##^*P* < 0.01 versus model group (TGF-*β*1).

By using the MTT method, we found that the viability of HSC in the model group was significantly increased when compared with the control group (*P* < 0.01). Different concentrations of AST reduced the cell viability, induced by TGF-*β*1, in a concentration-dependent manner (*P* < 0.01). The results were presented in Figure 1(a). In addition, comparing with the control group, the apoptosis rate in the model group was significantly reduced (*P* < 0.01), and AST promoted the apoptosis rates in a dose-dependent manner (*P* < 0.01). The results were shown in Figures 1(b) and 1(c). Additionally, LY-364947, as a positive control, could also inhibit cell viability and promote apoptosis in induced HSC, the same trend as AST.

### 3.2. AST Modulated ECM Factors

**Figure 2:**
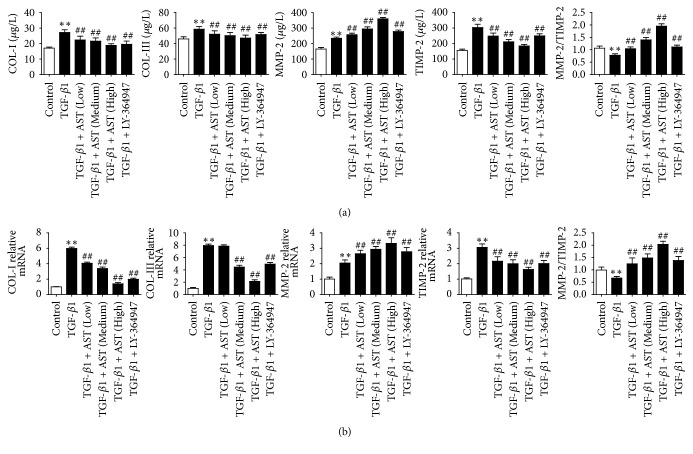
Effects of astragalosides on ECM factors in HSC induced by TGF-*β*1. By assay of ELISA, AST was found to inhibit collagen accumulation and the expression of MMP-2 and TIMP-2 (a), and the results of real-time PCR showed the same trend (b). AST: astragalosides; LY-364947: TGF-*β* receptor 1 inhibitor. The values presented are the means ± SD (*n* = 5 in each group). *P* < 0.05 and ^*∗∗*^*P* < 0.01 versus control group; *P* < 0.05 and ^##^*P* < 0.01 versus model group (TGF-*β*1).

Collagen is the major component of ECM and the contents of collagen I and collagen III increase significantly during hepatic fibrosis. In addition, typical fibrotic phenotype of the activated HSC is characterized by the imbalance of degradation and formation of ECM [18]. The results of ELISA showed that the protein levels of COL-I, COL-III, MMP-2, and TIMP-2 in HSC induced by TGF-*β*1 increased, while the ratio of MMP-2/TIMP-2 decreased when compared to the control group (*P* < 0.01), whereas AST at three different concentrations significantly decreased the levels of COL-I, COL-III, and TIMP-2, while the level of MMP-2 and the ratio of MMP-2/TIMP-2 increased compared to the model group in a concentration-dependent manner (*P* < 0.01). Additionally, LY-364947 showed the same trend as AST (Figure 2(a)). The results were also confirmed by the expressions of COL-I, COL-III, MMP-2, and TIMP-2 mRNA (Figure 2(b)).

### 3.3. AST Inhibited HSC Activation

**Figure 3:**
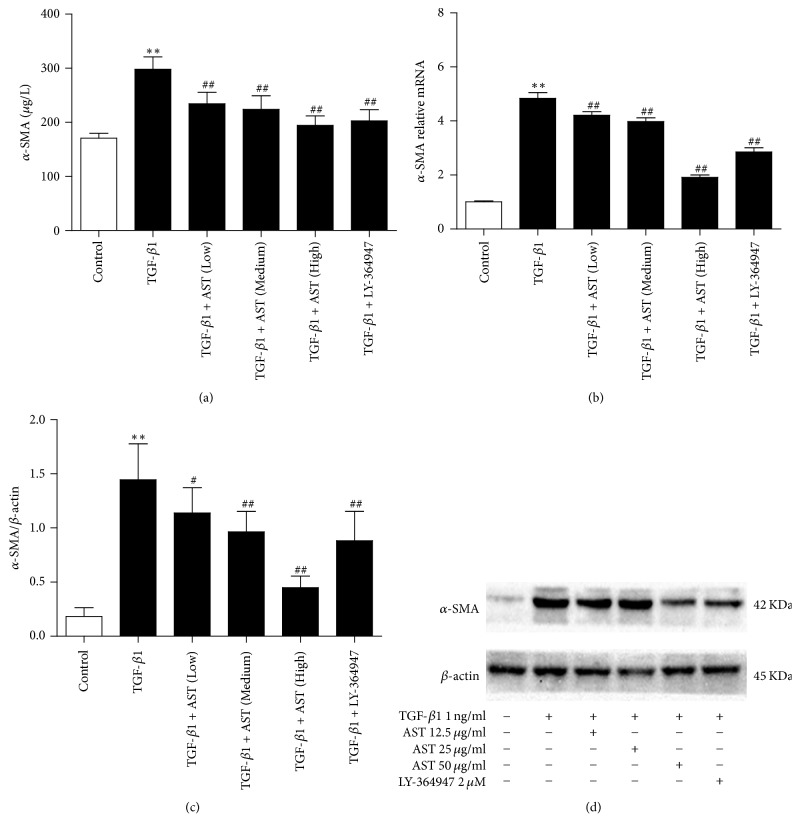
Astragaloside blocks the activation of HSC induced by TGF-*β*1. (a) By assay of ELISA, AST was found to decrease the expression of *α*-SMA. The expression of *α*-SMA was also assessed by real-time PCR (b) and western blot (c), normalized by *β*-actin. AST: astragalosides; LY-364947: TGF-*β* receptor 1 inhibitor. The values presented are the means ± SD (*n* = 5 in each group). *P* < 0.05 and ^*∗∗*^*P* < 0.01 versus control group; ^#^*P* < 0.05 and ^##^*P* < 0.01 versus model group (TGF-*β*1).

*α*-SMA was a marker of HSC activation, and its expression level is low in normal HSC. After being stimulated by TGF-*β*1, the expression of *α*-SMA increased significantly compared to the control group (*P* < 0.01). The results showed that AST could significantly downregulate the expression of *α*-SMA protein and mRNA in a dose-dependent manner. As expected, LY-364947 also shows the same effect (Figures 3(a), 3(b), and 3(c)).

### 3.4. AST Alleviated Liver Injury and Collagen Deposition in Rats

**Figure 4:**
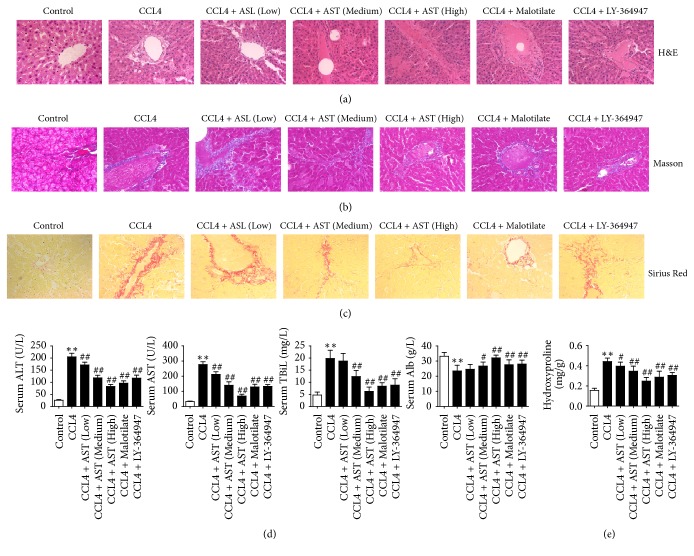
Inhibitory effects of AST on collage deposition and liver damage induced by CCL4 in rats. (a) The pathological features of liver tissue in each group were evaluated by H&E (×400), and collagen deposition was visualized by Masson's trichrome staining and Sirius red staining (×400) (b-c). AST was found to alleviate hepatic collagen accumulation and damage induced by CCL4. (d) Levels of ALT, AST, TBiL, and Alb in serum were detected by ELISA; AST was found to alleviate hepatic damage induced by CCL4 in a dose-dependent manner. (e) Hepatic fibrosis was evaluated by hydroxyproline content; AST was found to reduce the content of hydroxyproline by assay of ELISA. AST: astragalosides; LY-364947: TGF-*β* receptor 1 inhibitor. The values presented are the means ± SD (*n* = 10 in each group). *P* < 0.05 and ^*∗∗*^*P* < 0.01 versus control group; ^#^*P* < 0.05 and ^##^*P* < 0.01 versus model group (CCL4).

The histological damage and fibrosis of liver tissue were evaluated by H&E staining, Masson's trichrome staining, and Sirius red staining. Normal livers show a complete lobular architecture and no excess collagen deposition or necrosis was observed on the central vein; however, after CCL4 injection significant hepatic fibrosis, including hepatic lobular architecture destruction and pseudolobular and excessive collagen deposition, accompanied by necrosis, degeneration, and inflammatory infiltration was observed. Degeneration and necrosis were depressed in the AST group, with fewer complete pseudolobules and a small amount of fibrous tissues and inflammatory cells. Hepatic damage was also observed in the malotilate group and LY-364947 group (Figure 4(a)). In addition, Masson's trichrome staining and Sirius red staining showed that only a small amount of collagen existed in the walls of major blood vessels in normal liver. Administration of CCl4 caused dramatic accumulation of collagen. AST reduced collagen deposition in a dose-dependent manner (Figures 4(b) and 4(c)). Similar finding was found on the hydroxyproline content in liver tissue. Hydroxyproline content in rats increased after CCl4 treatment, while AST caused hepatic hydroxyproline content decrease (Figure 4(e)).

A significant increase in serum level of ALT, AST, and TBiL and a significant decrease of Alb level also confirmed hepatocellular injury induced by CCL4; these changes on serological indicators induced by hepatocellular injury were reversed by AST treatment; the same situation also appeared in the malotilate group and LY-364947 group (Figure 4(d)).

### 3.5. AST Decreased the Levels of Collagen-Related Indicators and *α*-SMA in Liver Tissues

**Figure 5:**
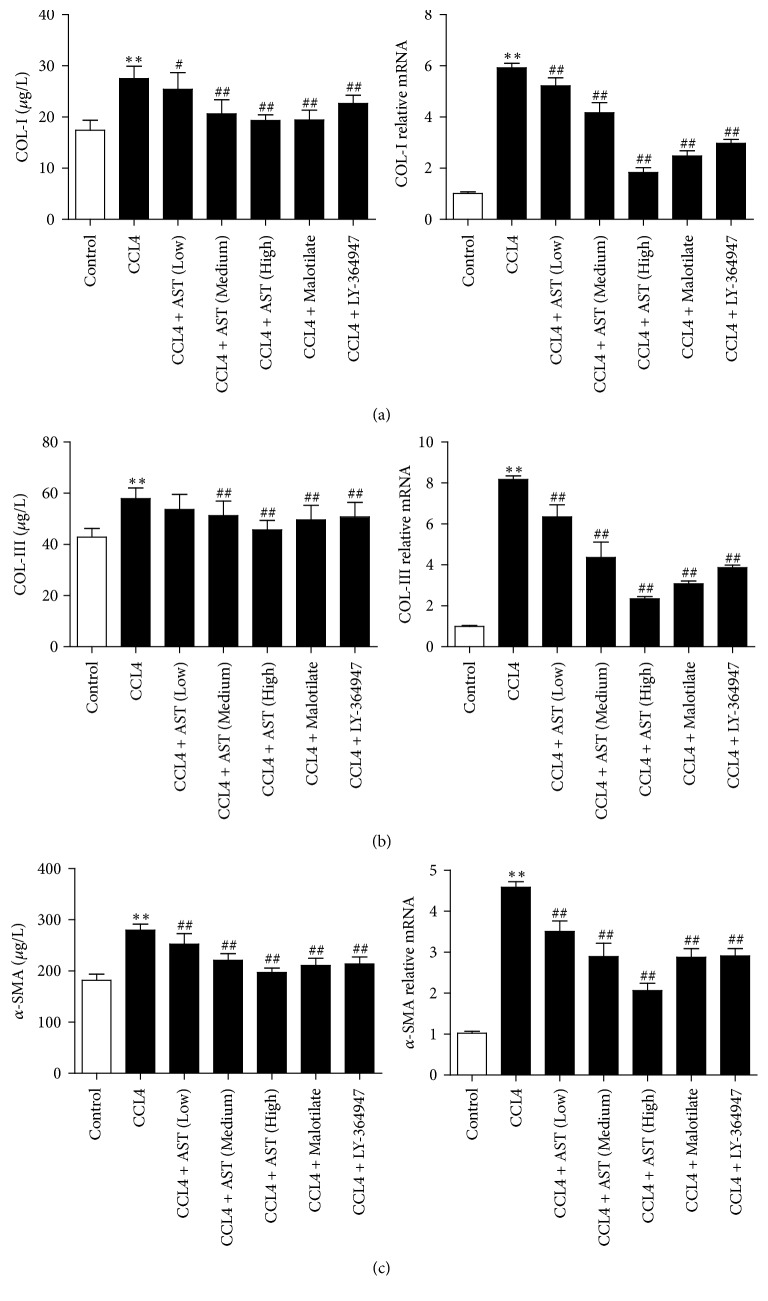
Astragalosides decreased the levels of collagen-related indicators and *α*-SMA in liver tissues. (a) By assay of ELISA and real-time PCR, AST was found to decrease the expression of COL-I induced by CCL4 in rats. (b-c) By assay of ELISA and real-time PCR, AST was also found to decrease the expression of COL-III and *α*-SMA. AST: astragalosides; LY-364947: TGF-*β* receptor 1 inhibitor. The values presented are the means ± SD (*n* = 10 in each group). *P* < 0.05 and ^*∗∗*^*P* < 0.01 versus control group; ^#^*P* < 0.05 and ^##^*P* < 0.01 versus model group (CCL4).

As shown in Figure 5, the contents of COL-I, COL-III, and *α*-SMA increased significantly in the model group (*P* < 0.01), which was reversed by AST treatment (*P* < 0.01). Similar change occurred on the expression of COL-I, COL-III, and *α*-SMA mRNA; this upregulation was inhibited by AST treatment group and other drugs (*P* < 0.01) (Figures 5(a), 5(b), and 5(c)).

### 3.6. AST Regulated the MDA, GSH, and SOD Levels in Liver Tissues

**Figure 6:**
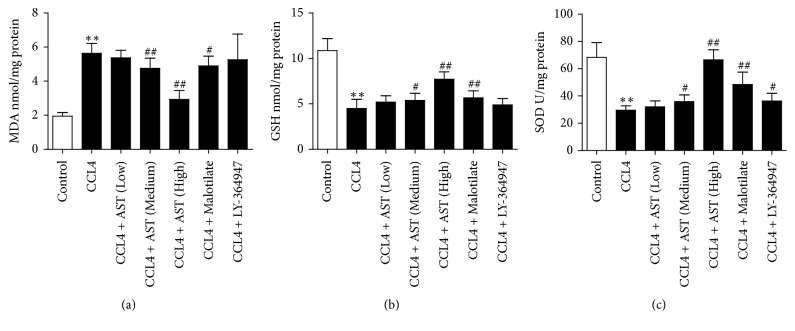
Effects of astragalosides on MDA, GSH, and SOD in liver tissues. (a) The content of malondialdehyde (MDA) was measured by thiobarbituric acid method; AST was found to increase the level of MDA. (b) The content of glutathione (GSH) was detected by spectrophotometry, and superoxide dismutase (SOD) was detected by xanthine oxidase method (c). AST was found to decrease GSH and SOD levels. AST: astragalosides; LY-364947: TGF-*β* receptor 1 inhibitor. The values presented are the means ± SD (*n* = 10 in each group). *P* < 0.05 and ^*∗∗*^*P* < 0.01 versus control group; ^#^*P* < 0.05 and ^##^*P* < 0.01 versus model group (CCL4).

Lipid peroxidation is closely related to hepatocyte injury and hepatic fibrosis. The level of MDA was increased dramatically while the levels of GSH and SOD were decreased significantly in liver tissue after CCL4 injection (*P* < 0.01). AST high dosage could significantly reverse the changes in MDA, GSH, and SOD levels caused by CCL4 (*P* < 0.01) (Figures 6(a), 6(b), and 6(c)).

### 3.7. AST Modulated the TGF-*β*1/Smad Signaling Pathway

**Figure 7:**
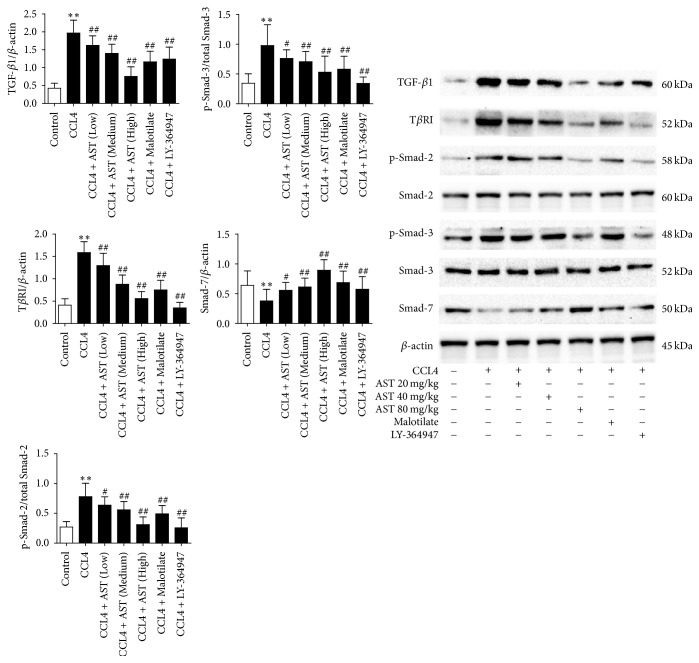
Effects of astragalosides on TGF-*β*_1_/Smad signaling pathway. The expressions of TGF-*β*_1_, T*β*R-I, p-Smad 2, Smad 2, p-Smad 3, Smad 3, and Smad 7 proteins were analyzed by western blot; Smad 2, Smad 3, and *β*-actin served as corresponding control. AST was found to attenuate the expression of TGF-*β*1, T*β*R-I, p-Smad 2, and p-Smad 3 proteins and enhance the expression of Smad 7, with no influence on the levels of Smad 2 and Smad 3 proteins. AST: astragalosides; LY-364947: TGF-*β* receptor 1 inhibitor. The values presented are the means ± SD (*n* = 10 in each group). *P* < 0.05 and ^*∗∗*^*P* < 0.01 versus control group; ^#^*P* < 0.05 and ^##^*P* < 0.01 versus model group (CCL4).

AST at three dosages could significantly inhibit the expression of TGF-*β*1 and T*β*R-I proteins (*P* < 0.01). Also, p-Smad 2 and p-Smad 3 were significantly increased in the liver tissue comparing to the control, with Smad 7 protein decreased significantly (*P* < 0.01). On the contrary, the expression of Smad 2 and Smad 3 proteins did not change. AST at three dosages could significantly inhibit the expression of p-Smad 2 and p-Smad 3 proteins and increase the expression of Smad 7 protein (*P* < 0.05 or *P* < 0.01), with no obvious effect on Smad 2 and Smad 3 proteins. The same pattern also appeared in malotilate treatment (*P* < 0.01). As an inhibitor of TGF-*β* receptor 1, LY-364947 was found significantly inhibiting the expression of T*β*R-I protein and the phosphorylation of its downstream Smad 2 and Smad 3 proteins (*P* < 0.01) (Figure 7).

**Figure 8:**
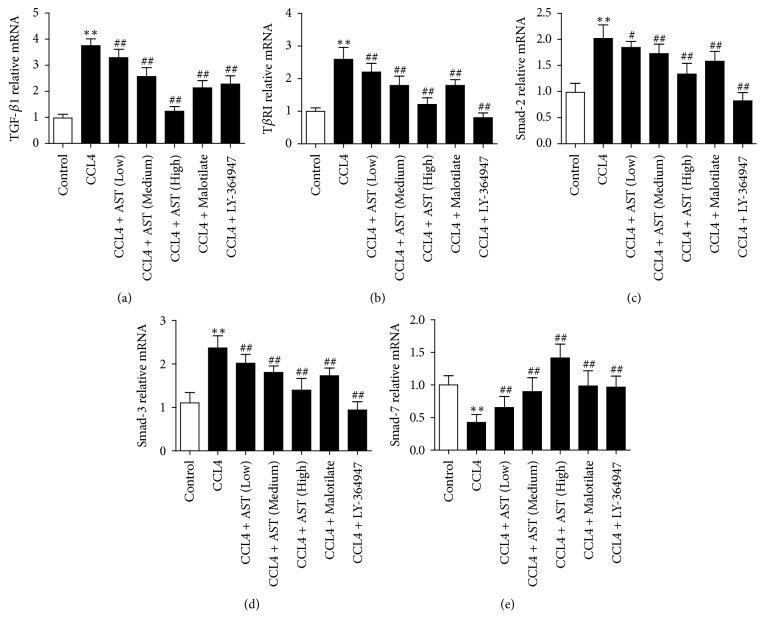
Effects of astragalosides on the mRNA expression of TGF-*β*1/Smad signaling pathway components in rats. The expression of TGF-*β*1, T*β*R-I, Smad 2, Smad 3, and Smad 7 mRNA was analyzed by real-time PCR, *β*-actin served as control, and the results were expressed as 2^−ΔΔCt^. AST was found to decrease the levels of TGF-*β*1, T*β*R-I, Smad 2, and Smad 3 mRNA, while the expression of Smad 7 was enhanced. AST: astragalosides; LY-364947: TGF-*β* receptor 1 inhibitor. The values presented are the means ± SD (*n* = 10 in each group). *P* < 0.05 and ^*∗∗*^*P* < 0.01 versus control group; ^#^*P* < 0.05 and ^##^*P* < 0.01 versus model group (CCL4).

The result was confirmed by real-time PCR, which showed a high expression of TGF-*β*1, T*β*R-I, Smad 2, and Smad 3 mRNA levels and a low expression of Smad 7 mRNA in model group, respectively, in comparison with the control (*P* < 0.01). On the other hand, the expression of TGF-*β*1, T*β*R-I, Smad 2, and Smad 3 mRNA decreased with Smad 3 mRNA increased obviously in the treatment groups, especially with AST at high dosage (*P* < 0.01) (Figure 8).

### 3.8. Immunohistochemical Analysis of AST on TGF-*β*1/Smad Signaling

**Figure 9:**
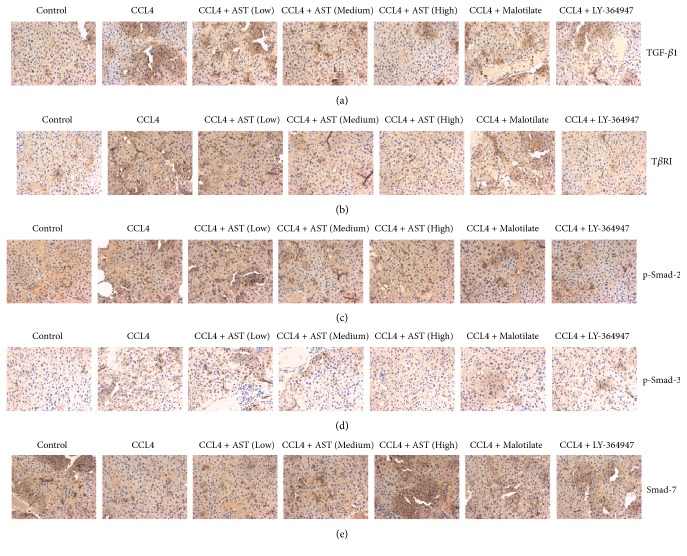
Effects of astragalosides on TGF-*β*1/Smad signaling induced by CCL4 in rats. The expressions of TGF-*β*1, T*β*R-I, p-Smad 2, p-Smad 3, and Smad 7 proteins were analyzed by immunohistochemistry. AST was found to decrease the amounts of TGF-*β*1, T*β*R-I, p-Smad 2, and p-Smad 3 proteins, while the content of Smad 7 protein was increased. AST: astragalosides; LY-364947: TGF-*β* receptor 1 inhibitor. The values presented are the means ± SD (*n* = 10 in each group).

Immunohistochemical results showed that TGF-*β*1 and T*β*R-I proteins were expressed at very low level in the normal rats. After CCL4 injection, fibrosis occurred in liver tissue as well as a large amount of TGF-*β*1 and T*β*R-I positive expression around the fibrous septum. AST could significantly downregulate the expression of TGF-*β*1 and T*β*R-I proteins in liver tissue. The same effect also appeared in the malotilate and LY-364947 groups, while LY-364947 is known to inhibit the expression of the T*β*R-I protein (Figures 9(a) and 9(b)). In the meantime, p-Smad 2 and p-Smad 3 were weakly expressed in the normal tissue. The expression of p-Smad 2 and p-Smad 3 increased in hepatocytes around the fibrotic region and its periphery after CCL4 injection. The expression was mainly found in the nucleus. AST could significantly inhibit the phosphorylation of Smad 2 and Smad 3, especially for the phosphorylation of Smad 2. The same remediation also occurs in the malotilate and LY-364947 groups (Figures 9(c) and 9(d)). In addition, the expression of Smad 7 protein was significantly low in the model rats. AST, malotilate, and LY-364947 could promote the expression of Smad 7 protein and the effect of AST at high dosage was most obvious (Figure 9(e)).

## 4. Discussion

TGF-*β* superfamily is a group of multifunctional polypeptides, including TGF-*β*, bone morphogenetic protein, inhibin, and activin. TGF-*β*, closely related to cirrhosis, is composed of 5 members, TGF-*β*1 to TGF-*β* 5, among which only TGF-*β*1 is associated with hepatic fibrosis [19]. As the most important virulence factor of hepatic fibrosis, TGF-*β*1 is widely found in normal tissues and transformed cells of animals, with the most abundant content in bone tissues and platelets [20]. It can inhibit the proliferation, induce cell differentiation and immunosuppression, promote ECM synthesis, and regulate collagen production and tissue repair. Its concentration is low in normal liver but increases significantly when liver injury occurs. Parenchymal cells, Kupffer cells, sinusoidal endothelial cells, fibroblasts, platelets, and other migrating inflammatory cells can release large amounts of TGF-*β*1 when induced by various pathogenic factors during hepatic fibrosis [21]. Moreover, activated HSC eventually becomes the major source of TGF-*β*1 production, forming a positive feedback loop [22]. The current experiment in vitro also confirmed that HSC could be activated by TGF-*β*1, and AST could inhibit the expression of a-SMA in HSC, thereby inhibiting cell activation and promoting apoptosis.

TGF-*β*1/Smad signaling pathway consists of three parts, extracellular TGF-*β*1, the membrane-bound TGF-*β*1 receptor (T*β*R), and intracellular Smad proteins. There are at least 5 different types of TGF-*β*1 receptors (I-V), where T*β*R-I and T*β*R-II are signal transduction receptors, which are necessary for signal transduction. T*β*R belongs to the transmembrane serine/threonine kinase receptors; in the absence of ligands, they remain as dimers. Once TGF-*β*1 binds to its receptor, T*β*R-I and T*β*R-II are activated and then catalyze the phosphorylation of the serine residue on the downstream Smad molecule. Finally, phosphorylated Smad enters the nucleus and regulates transcription of target genes related to hepatic fibrosis. Smad is the only known intracellular substrate of type I receptor. Smad family proteins include at least nine Smad proteins, expressed as Smad l-9, which are divided into receptor-activated Smad (R-Smad), common pathway Smad (Co-Smad), and inhibitory Smad (I-Smad). R-Smad, such as Smad 2 and Smad 3, are mainly known to promote liver fibrosis, whereas I-Smad such as Smad 7 can inhibit or regulate the TGF-*β* family of signal transduction and inhibit hepatic fibrosis. Since TGF-*β*1/Smad signaling pathway plays such an important role in hepatic fibrosis, blocking or mediating the signal transduction is an important potential strategy to prevent and treat hepatic fibrosis [23, 24]. Ly-364947 is a pyrazole-based small molecule that acts as a competitive binding inhibitor of ATP. It inhibits p-Smad 3 phosphorylation by inhibiting to T*β*R-I kinase [25]. In vivo results showed that CCL4 treatment could increase the expression of TGF-*β*1, T*β*R-I, Smad 2, and Smad 3 in liver tissue and inhibit the expression of Smad 7, thereby reducing the formation of liver fibrosis. In the present study, we found that AST downregulated p-Smad 2 and p-Smad 3 and upregulated Smad 7, thereby inhibiting the TGF-*β*/Smad signaling pathway. That is the mechanism by which AST suppresses CCl4-induced hepatic fibrosis.

Imbalance between oxidative and antioxidant systems due to damage stimulus that leads to inflammation, activation of HSC, proliferation, and secretion of extracellular matrix has been confirmed to play an important role in the formation and development of hepatic fibrosis [26–28]. MDA is the final product in lipid peroxidation. Therefore, the content of MDA reflects the degree of lipid peroxidation, which indirectly represents the level of oxidative injury [29]. As important antioxidant enzyme and free radical scavenger, SOD and GSH can remove the harmful free oxygen radicals [30]. Our in vivo results showed that AST could eliminate oxidative stress and hepatocyte injury by attenuating the levels of MDA and enhancing SOD and GSH productions. This must contribute to the remediation of hepatic fibrosis as previous studies suggested [31, 32].

MMPS family is a cluster of zinc dependent proteases. Their main function is to degrade ECM that plays a key role in physiological and pathological processes. MMP-2 (gelatinase A) is a type of collagenase that involves the metabolism of type IV collagen in the basement membranes and hereby closely plays a role in the development of hepatic fibrosis. TIMP-2, an endogenous inhibitor of MMP-2 in the tissue, can bind to MMP-2 at 1 : 1 ratio to form a TIMP-2/MMP-2 heterodimer and hence modulate the proteolytic activity of MMP-2. In turn, TIMP-2 can bind to the C-terminus of gelatinase A to form an inhibitory complex in the cell. This complex interacts with TIMP-1-MMP-2 heterodimer to form a heterotrimer to activate gelatinase A. Excessive TIMP-2 inhibits the activation of gelatinase A, but by the formation of a three-molecule complex the presence of an appropriate amount of TIMP-2 appears to be essential for the activation of gelatinase A. Both MMP-2 and TIMP-2 play a role in maintaining the steady state of the liver cell microenvironment; once the balance is broken by pathogenic factors, hepatic fibrosis occurred [33]. This study showed that MMP-2 and TIMP-2 levels increased in activated HSC and the ratio of MMP-2/TIMP-2 decreased. AST could increase the level of MMP-2 and decrease the level of TIMP-2 in HSC, thus restoring the ratio of TIMP-2/MMP-2 and finally promoting the degradation of COL-I and COL-III in the cells and liver tissues.

In conclusion, AST is an effective antifibrosis ingredient extracted from Radix* Astragali*. This is the first time its mechanism in hepatocellular protection and hepatic fibrosis mediation has been demonstrated. AST can inhibit the activation of HSC and promote cell apoptosis. Additionally, AST can also regulate the expression of MMP-2/TIMP-2, which may promote the synthesis and metabolism of collagen and facilitate the degradation of ECM. It also regulates lipid peroxidation and downregulates TGF-*β*1, T*β*RI, p-Smad 2, and p-Smad 3 mRNA expression and upregulates Smad 7 mRNA in the TGF-*β*1/Smad signaling pathway. Therefore, AST is a potential clinical drug for treating hepatic fibrosis.

## Figures and Tables

**Table 1:** Primers used in real-time PCR reactions.

	Forward	Reverse	Products (bp)
COL-I	TTTGGGATTGGCTGGTTAGATTAG	AACAAACAGGACTCAGGAGGGTAAA	169
COL-III	GTGCTGAAGGGCAGGGAACA	CGGTGAAGCAGGGTGAGAAGA	148
*α*-SMA	CCCTGAAGTATCCGATAGAACACG	CCATCTCCAGAGTCCAGCACAAT	277
MMP-2	GGCAGAATGTGGAAACAGTC	GGGCTAAATTCATGGGTTCC	176
TIMP-2	GTTGTTGCTGTGGCTGATAG	TGTGGGACCTGTGGAAGTA	266
TGF-*β*1	GAACCAAGGAGACGGAATACAGG	ACCTCGACGTTTGGGACTGATC	113
T*β*R-I	GAAATCGCTCGACGCTGTTC	TTCGCAAAGCTGTCAGCCTA	239
Smad 2	CACTGCTGACGGACTTTAGGACAT	ATACCGGAGGCAGACAGTAACAAG	144
Smad 3	CCACGACTGCCCTTGTTGC	GCTGGTGAGAACCGCTTCTTC	135
Smad 7	CCGATCTTGCTCCTCACTTTCTG	CACTGGTGCGTGGTGGCATAC	177
*β*-Actin	GCGAGAAGATGACCCAGATCATGTT	GCTTCTCCTTAATGTCACGCACGAT	300
